# Upregulated Expression of SHMT2 Predicts Poor Survival of Lung Adenocarcinoma

**DOI:** 10.1155/genr/6104753

**Published:** 2025-05-18

**Authors:** Qi Guo, Guang-Hong Huang, Pu Chen, Chao Guo

**Affiliations:** ^1^Department of Surgery, Xi'an Chest Hospital, Xi'an 710000, China; ^2^Department of Anesthesiology and Surgery, Xi'an Chest Hospital, Xi'an 710000, China

**Keywords:** adenocarcinoma of the lung, biomarkers, prognosis, serine hydroxy methyltransferase 2, survival analysis

## Abstract

**Backgrounds:** Serine hydroxy methyltransferase 2 (SHMT2) exerts an essential function in the cellular serine/glycine biosynthesis and one-carbon metabolism. Accumulative evidence revealed that SHMT2 was involved in cancer initiation and development in several types of carcinomas such as glioma, intrahepatic cholangiocarcinoma and colorectal cancer. However, expression and role of SHMT2 in lung adenocarcinoma (LUAD) had not been fully investigated.

**Methods:** Transcriptional information of SHMT2 was retrieved from TCGA database. mRNA and protein expression of SHMT2 were analyzed in LUAD tissues alongside adjacent normal lung tissues using quantitative RT-PCR and immunohistochemical staining. The prognostic significance of SHMT2 in LUAD was assessed through both univariate and multivariate statistical analyses.

**Results:** SHMT2 was higher in LUAD tissues than that in adjacent lung tissues on transcriptional level, mRNA level, and protein level. Elevated SHMT2 protein levels were associated with increased tumor size, positive lymph node metastasis, and more advanced TNM stages. LUAD patients with high SHMT2 level had worse prognosis.

**Conclusions:** Our research indicated that elevated SHMT2 expression is strongly linked to adverse clinical characteristics and poor prognosis in LUAD patients. Consequently, SHMT2 may represent a novel prognosis marker and a promising therapeutic target regarding the treatment of LUAD.

## 1. Introduction

Lung adenocarcinoma (LUAD) is a subtype of non-small-cell lung cancer (NSCLC), representing 40% of lung cancers [1]. LUAD is one of the most common malignancies among nonsmokers and is a leading reason of cancer-related deaths [2]. Present treatment choices for LUAD patients include surgery treatment, chemotherapy, radiation therapy, targeted therapies, immunotherapy, etc. [3–6]. Despite significant advancements in these treatment methods, the 5 year survival rate for LUAD cases remains below 16%, largely because of the challenges of early diagnosis [7–9]. Thus, exploring the oncogenic mechanism underlying LUAD and classifying possible therapeutic targets is crucial for developing more effective treatments and improving patient prognosis.

Metabolic reprogramming in cancer cell is important for their survival and proliferation [10]. One of the most notable metabolic changes is the folate-dependent one-carbon (1C) metabolism [11]. It is a widespread metabolic process that serves to catalyze and transfer 1C units for biosynthetic processes such as nucleotide synthesis, methylation reactions and redox balance [12, 13]. Therefore, one-carbon metabolism plays a crucial role in maintaining cellular homeostasis, which provides cells with several components including lipids, nucleotides and proteins, and is also essential in supporting tumor growth [14, 15]. The role of one-carbon metabolism in cancer pathogenesis has been extensively explored, and some antagonists of one-carbon metabolic enzymes have been used as frontline chemotherapy for cancer treatment [16, 17].

Serine hydroxy methyltransferase (SHMT) plays a critical role in cellular one-carbon metabolism by catalyzing the formation of glycine [18]. There are two isoforms of SHMT proteins in eukaryotes: the cytoplasmic isoform (SHMT1) and the mitochondrial isoform (SHMT2) [19]. SHMT2 contributes a lot in the mitochondrial thymidylate biosynthesis and one-carbon pathway [20]. A recent study showed that SHMT2 played a crucial role in carcinogenesis and cancer development within several types of carcinoma, such as glioma, intrahepatic cholangiocarcinoma and colorectal cancer [21–23]. Highly expressed SHMT2 was found to be correlated with the worse clinical features in triple-negative breast cancer patients [24]. Besides, knockdown of SHMT2 significantly inhibit tumor proliferation [25]. And some evidences showed that SHMT2 overexpression supports cancer cell survival in melanoma cells [26]. However, the clinical significance of SHMT2 in human LUAD has yet to be established.

To assess the clinical role of SHMT2 in LUAD patients, we firstly retrieved its transcriptional level from TCGA database, then detected mRNA and protein levels of SHMT2 in LUAD together with adjacent lung. Next, we examined the relationship between SHMT2 levels and survival outcomes of LUAD cases.

## 2. Materials and Methods

### 2.1. Database Analysis

We conducted a comprehensive analysis of SHMT2 transcriptional levels and their prognostic significance in LUAD using publicly available databases. GEPIA (https://gepia.cancer-pku.cn/index.html), an interactive tool for RNA sequencing data from TCGA and GTEx projects, was utilized to compare SHMT2 expression levels between 59 normal lung tissues and 483 LUAD samples. Expression levels were quantified in transcripts per million (TPM), and statistical tools within GEPIA were applied to assess differential expression and its association with tumor stage. To evaluate the prognostic impact of SHMT2, we employed both GEPIA and the Kaplan–Meier plotter database (https://www.kmplot.com). Overall survival (OS) was analyzed using GEPIA, while OS and progression-free survival (PFS) were examined with the Kaplan–Meier plotter. Patients were categorized into high- and low-expression groups based on the median SHMT2 level, and survival outcomes were compared using log-rank tests.

### 2.2. Patients and Specimens

The Ethics Committee of Xi'an Chest Hospital approved this study (Protocol No. 2022-05-0028), and written informed consent was obtained from all enrolled patients. We randomly selected 113 formalin-fixed LUAD samples along with adjacent normal lung tissues from LUAD cases at Xi'an Chest Hospital during 2015-2016. The inclusion criteria were: (1) histopathological diagnosed with LUAD at Xi'an Chest Hospital between 2015 and 2016 by a qualified pathologist; (2) availability of formalin-fixed LUAD tissue samples along with adjacent normal lung tissue for analysis; (3) patients with complete clinical and follow-up data, including survival information; (4) follow-up duration of at least 12 months. Exclusion criteria were: (1) patients with incomplete or missing clinical or follow-up data; (2) Patients diagnosed with secondary malignancies or other coexisting cancers during the study period; (3) Cases with prior neoadjuvant therapy or other treatments that could interfere with the analysis of tumor characteristics. Finally, all these 113 LUAD patients were followed for 12–108 months, with 37 participants succumbing to cancer by the end of the follow-up period. The median survival time for the cohort was 72 months, and the 5 year survival rate was 69.6%. All experiment samples tested in our research has been validated through histopathology examination.

### 2.3. RNA Extraction and qRT-PCR

Additionally, 27 consecutive fresh-frozen LUAD specimens and corresponding normal lung samples were collected during March 2023 to April 2023, for further qRT-PCR analysis. For mRNA analysis, we firstly extracted mRNA from experiment samples. Subsequently, isolated mRNAs were treated with DNase and reversely transcribed into cDNA based on the reverse transcription kit (Thermo Fisher Scientific, USA). Then RT-qPCR was conducted with the use of SYBR Green PCR Master Mix (Thermo Fisher Scientific, USA). The primers' sequences were listed below:

  SHMT2-Forward: 5′-CGAGTTGCGATGCTGTACTT-3′
  SHMT2-Reverse: 5′-CTGCGTTGCTGTGCTGAG-3′
  GAPDH-Forward: 5′-GTCTCCTCTGACTTCAACAGCG-3′
  GAPDH-Reverse: 5′-ACCACCCTGTTGCTGTAGCCAA-3′

### 2.4. Immunohistochemistry (IHC) Staining

IHC staining targeting SHMT2 was performed according to manufacturer instructions for the 113 cases with formalin-fixed LUAD samples. The fixed samples were firstly cut into 4 μm serial sections and then dried and deparaffinized. Next step, the sections were taken for microwave antigen retrieval within citrate buffer (pH 6.0). After further blocking and incubating with anti-SHMT2 antibody (1 : 500 dilution; Cat. No. NBP2-20354; Novus Biologicals) at 4°C overnight, slide sections were treated with poly HRP IgG and 3,3′-diaminobenzidine substrates to visualize the immunoreactive signals. Negative control was conducted by replacing primary antibody with PBS.

### 2.5. Evaluation of IHC Staining

We randomly selected eight fields per sample for observation. The IHC staining intensity was classified as: 1 (negative), 2 (weak), 3 (moderate), and 4 (strong). Percentage of positively stained cells was classified as: 1 (0%–25%), 2 (26%–50%), 3 (51%–75%), and 4 (76%–100%). The total IHC score was calculated by multiplying the two scores above. To further assess clinical significance of SHMT2 in LUAD, cases were subgrouped as two groups based on the IHC score: high-SHMT2 group (score ≥ 5, *N* = 56) and low-SHMT2 group (score < 5, *N* = 57).

### 2.6. Statistical Analysis

Statistical analysis was performed using SPSS Statistics 20.0. Associations between SHMT2 protein levels and adverse clinical outcomes were assessed by Chi-square test. Kaplan–Meier analyses were employed to plot the OS curves of the LUAD patients. Independent prognosis factors were classified through multivariate analysis. A *p* value of less than 0.05 was believed statistically significant.

## 3. Results

### 3.1. The Transcriptional Level of SHMT2 and Its Prognostic Significance in LUAD

**Figure 1:**
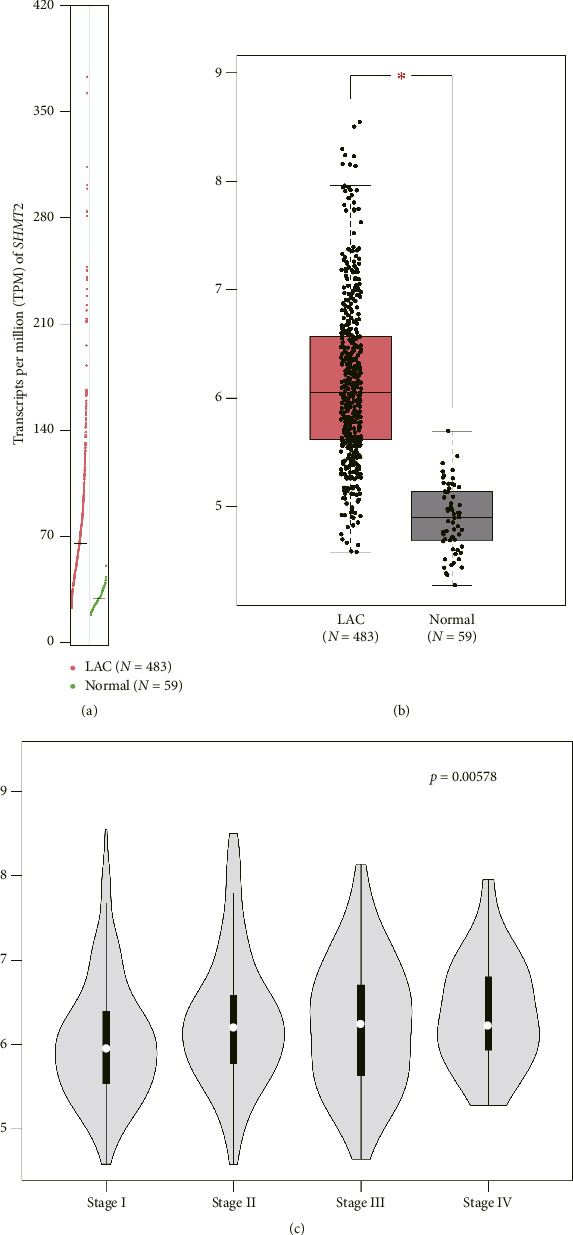
Transcriptional level of SHMT2 from GEPIA database. (a) The transcriptional level of SHMT2 was obtained from GEPIA (https://gepia.cancer-pku.cn/index.html) interactive online server, including 59 normal lung tissues and 483 lung adenocarcinoma tissues. (b) By plotting the box plot, a higher level of SHMT2 transcription was observed in lung adenocarcinoma tissues compared to normal lung tissues. (c) The transcriptional difference of SHMT2 in LUAD tissues from various TNM stages was compared, which showed that tissues with more advanced stages possessed higher SHMT2 transcriptional level.

Here we firstly extracted transcriptional level of SHMT2 using GEPIA (https://gepia.cancer-pku.cn/index.html) interactive online server [27]. After analyzing 59 normal lung tissues and 483 LUAD tissues according to TCGA and GTEx database, a higher level of TPM (transcripts per million) of SHMT2 was observed in LUAD specimens compared to normal lung specimens (Figures 1(a), 1(b)). Of note, comparing to LUAD tissues with earlier clinical stage, SHMT2 possessed more transcripts in those with advanced TNM stage, indicating its positive correlation with tumor stages in LUAD patients (Figure 1(c)).

**Figure 2:**
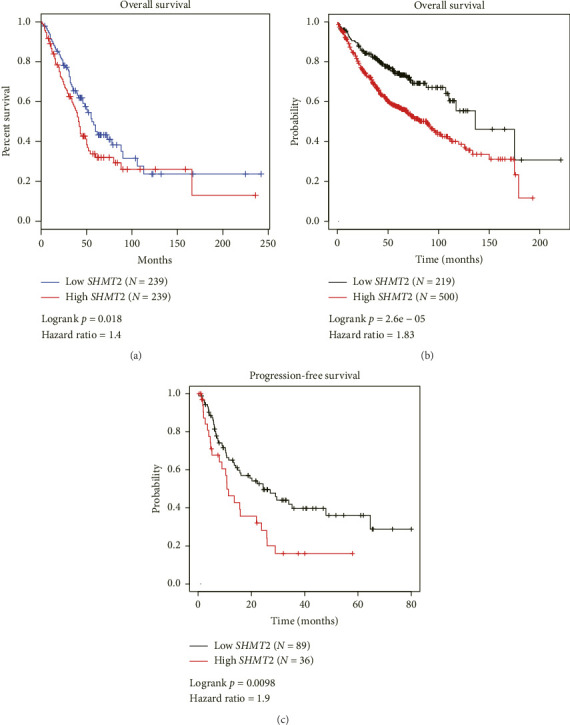
High SHMT2 transcription indicates poor survival of LUAD patients. (a) The prognostic effect of SHMT2 transcription was analyzed from GEPIA database (https://gepia.cancer-pku.cn/index.html), indicating its unfavorable predictive role on LUAD overall survival. (b), (c) K-M plotter database (https://www.kmplot.com) was also utilized to provide additional evidence, which confirmed that higher SHMT2 transcription was correlated to poorer overall survival and progression-free survival of LUAD patients.

We also analyzed the effect of SHMT2 transcriptional level on patients' survival using both GEPIA (Figure 2(a)) and K-M plotter database (Figures 2(b), 2(c); https://www.kmplot.com). Accordingly, patients with higher SHMT2 transcriptional level exhibited shorter OS time (Figures 2(a), 2(b)) as well as shorter PFS time (Figure 2(c)). Therefore, enhanced SHMT2 transcription may be closely correlated with unfavorable prognosis of LUAD patients.

### 3.2. The mRNA and Protein Levels of SHMT2 in LUAD

**Figure 3:**
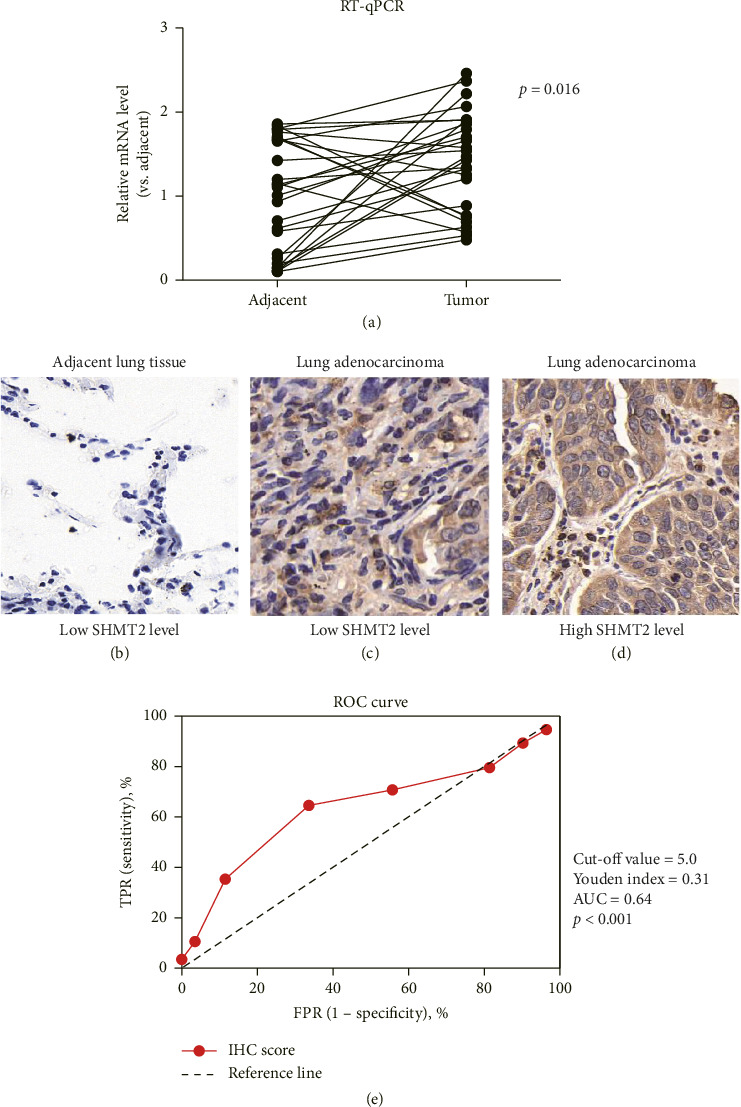
Analyses of mRNA and protein expression of SHMT2 in LUAD patients. (a) mRNA levels of SHMT2 were assessed in 27 pairs of fresh-frozen LUAD tissues and adjacent normal lung tissues using real-time qPCR. (b), (c), (d) IHC staining results displayed representative high expression levels of SHMT2 (d) and representative low expression levels (b), (c) in LUAD and adjacent lung tissues. Magnification, 400x. ^∗^, *p* < 0.05 by student's *t*-test. (e) The ROC curve was generated to determine the cut-off value for the IHC score.

To systematically examine expression pattern of SHMT2 in LUAD, we measured mRNA levels of SHMT2 in 27 pairs of fresh LUAD samples and adjacent normal lung specimens using qRT-PCR. Our data indicated that the mRNA level of SHMT2 was significantly higher in LUAD specimens compared to normal lung specimens (Figure 3(a)).

In addition, our data examined protein level of SHMT2 in an additional cohort containing 113 cases of fixed LUAD specimens together with adjacent normal lung tissues by IHC method (Figures 3(b), 3(c), 3(d)). IHC data demonstrated that protein level of SHMT2 was significantly higher in LUAD specimens than that in adjacent control specimens. Taken together, these data indicated that mRNA and protein level of SHMT2 was upregulated in LUAD samples, which is consistent with its transcriptional level as retrieved from TCGA database.

### 3.3. Associations Between SHMT2 Protein Expression and Patients' Characteristics

To understand the association between SHMT2 expression levels and clinical prognoses of those cases, all enrolled cases were subgrouped into two groups as described above. ROC curve was carried to determine cut-off value of IHC scores (Figure 3(e)). Next we assessed relationships between SHMT2 and unfavorable clinicopathological characteristics of LUADs (Table 1). And data showed that high expression of SHMT2 was significantly associated with bigger tumor size (*p*=0.009), positive lymph nodes (*p*=0.011) and advanced TNM stages (*p*=0.002). However, no significant associations were found between SHMT2 protein levels and patients' age, gender, tumor differentiation, or smoking history (*p* > 0.05).

### 3.4. High SHMT2 Is Correlated With Poor Survival of LUAD

**Figure 4:**
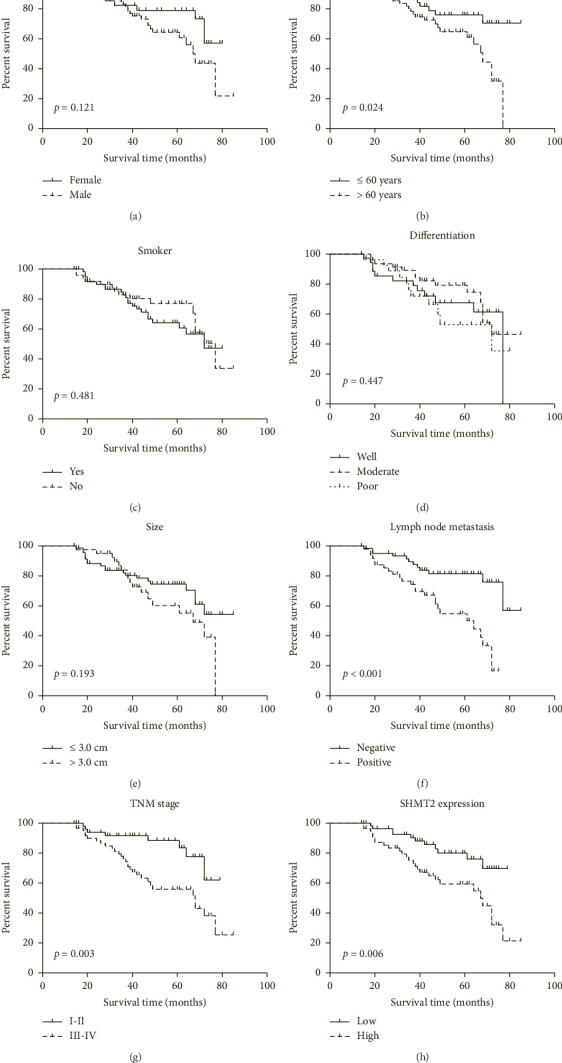
Overall survival analyses of our enrolled cohort. Overall survival curves for LUAD patients were plotted using Kaplan–Meier analysis and assessed via log-rank test, based on patients' gender (a), age (b), smoking history (c), tumor differentiation (d), tumor size (e), lymph node metastasis (f), TNM stage (g), and SHMT2 expression level (h), respectively.

Next, our study examined relationship between patients' characteristics and OS in LUAD patients using the Kaplan–Meier method and log-rank test (Figures 4(a), 4(b), 4(c), 4(d), 4(e), 4(f), 4(g)). The results also revealed that LUAD patients with higher SHMT2 expression had a significantly poorer mean OS time (57.9 ± 3.6 months) compared to those with lower SHMT2 expression (68.9 ± 2.7 months; *p*=0.006; Figure 4(h)). Additionally, other clinicopathological factors, such as age, lymph node metastasis, and TNM stage, were also found to be associated with OS (all *p* < 0.05; Figure 4, Table 2).

Subsequently, we assessed their independent effects on the OS time of LUAD patients using the Cox regression test (Table 3). Specifically, we did not include LN status in the multivariate analysis due to its close correlation with the TNM stage, despite its significance in the univariate analysis. This approach was taken to avoid multicollinearity issues, ensuring the robustness of the multivariate model. As a result, SHMT2 protein expression was identified as an independent prognostic factor (hazard ratio = 2.120, 95% confidence interval = 1.027–4.376, *p*=0.042). Additionally, age (*p*=0.014) and TNM stage (*p*=0.014) were also recognized as independent factors associated with poor survival in LUAD patients.

## 4. Discussion

Tumor cell survival and proliferation depends on their metabolic changes, such as aerobic glycolysis, enhanced lipid uptake and folate-dependent one-carbon metabolism [28]. The enzyme SHMT is required to catalyze the conversion of serine and tetrahydrofolate (THF) into glycine and 5,10-methylene–THF, respectively [29]. Elevations in the synthesis and utilization of serine and glycine have been observed in transformed cells [30]. Cumulative evidence has indicated the critical function of serine and glycine in promoting oncogenesis [11]. Glycine is crucial for maintaining cellular homeostasis and supporting oxidative phosphorylation in mitochondria [12]. Previous studies have indicated that glycine uptake and metabolism may promote tumorigenesis and malignancy, highlighting serine/glycine metabolism as a potential novel target for therapeutic intervention [16].

SHMT2 plays a pivotal role in one-carbon metabolism, a crucial cellular process involving the transfer of one-carbon units for biosynthetic reactions. Specifically, SHMT2 catalyzes the conversion of serine to glycine, generating 5,10-methylenetetrahydrofolate, which is essential for nucleotide synthesis. This process is vital for DNA replication and repair, making SHMT2 indispensable for rapidly proliferating cells, such as cancer cells. Additionally, SHMT2 contributes to maintaining redox balance by producing NADPH, a key reducing agent that protects cells from oxidative stress. This redox regulation is crucial for cell survival under stressful conditions, such as those encountered in the tumor microenvironment. SHMT2 also impacts epigenetic regulation by supplying methyl groups necessary for DNA and histone methylation, thus influencing gene expression patterns involved in cancer progression.

SHTM2 is an important enzyme which contributes a lot to the serine/glycine metabolism pathway [31]. Its gene located on chromosome 12 and encoded the 52 kD protein SHMT2 which is mainly expressed in the mitochondria [10]. Its cytosolic isoform SHMT1 has been widely studied and was reported in progression of various cancer types [18]. It has been recently reported that a small-molecule dual inhibitor of human SHMT1/2 could inhibit cancer cell survival through suppressing glycine demand, especially in diffuse large B-cell lymphoma [32]. Moreover, oncogenomic studies indicated that SHMT2 might function as a potential oncogene [7]. Accumulative evidence also showed that SHMT2 was highly overexpressed in several cancers than normal tissues and it could promote tumor cell initiation and progression through regulating serine/glycine metabolism [33]. However, the potential effect of SHMT2 in human LUAD remains poorly understood. The aim of our research is therefore to explore clinical role of SHMT2 in LUAD patients, given its important function in serine/glycine metabolism pathway.

Recent studies have highlighted SHMT2's role in promoting tumor cell proliferation, migration, and resistance to apoptosis. For instance, research has shown that overexpression of SHMT2 is correlated with enhanced proliferation and invasion capabilities in different cancers, including LUAD. Furthermore, SHMT2 has been implicated in conferring resistance to apoptosis by modulating the cellular redox state, thereby enabling cancer cells to evade programmed cell death mechanisms. The findings underscored the capacity of SHMT2 as a therapeutic target, as inhibiting its activity could disrupt these critical pathways, reducing tumor growth and metastasis. Future research should focus on elucidating the precise molecular mechanisms by which SHMT2 regulates these processes and exploring SHMT2 inhibitors' therapeutic efficacy in preclinical and clinical settings.

In our study, we reported that both mRNA and protein levels of SHMT2 were remarkably upregulated in LUAD specimens comparing to adjacent lung specimens, as shown by qRT-PCR and IHC staining. To further assess clinical significance of SHMT2 in LUAD, we categorized the cohort into two subgroups based on IHC result. We also explored relationships between high SHMT2 level and negative prognosis, finding that elevated SHMT2 levels were significantly associated with larger tumor size, positive lymph node metastasis, and advanced TNM stage. Additionally, multivariate analysis validated SHMT2 level as an independent prognostic factor for LUAD patients.

However, our data predominantly focused on the clinical implication and phenotype effects of SHMT2 on LUAD without illuminating its functional mechanism. Therefore, further research aimed at uncovering the molecular mechanisms by which SHMT2 promotes LUAD progression will be essential for providing more evidence on its therapeutic potential.

## 5. Conclusion

Taken together, our data showed SHMT2 expression levels were upregulated in LUAD specimens and significantly related with poorer survival in LUAD cases. This study highlights the critical function of SHMT2 in LUAD and suggests that it may act as a possible prognosis predictor and therapeutic target for the disease.

## Figures and Tables

**Table 1:** Characteristics of the LUAD patients and associations with SHMT2 expression level.

Variable	Cases (*n* = 113)	SHMT2 expression		*p* value
		Low (*n* = 57)	High (*n* = 56)	
Sex				0.790
Female	37	18	19	
Male	76	39	37	
Age (years)				0.515
≤ 60 years	49	19	21	
> 60 years	64	22	19	
Smoker				0.933
No	50	19	15	
Yes	63	22	25	
Differentiation				0.643
Well	39	31	18	
Moderate	47			
Poor	27	10	22	
Tumor size				0.009^∗^
≤ 3.0 cm	72	15	16	
> 3.0 cm	41	26	24	
LN metastasis				0.011^∗^
Negative	64	27	15	
Positive	49	14	25	
TNM stage				0.002^∗^
I-II	53	30	20	
III-IV	60	11	20	

*Note:* LUAD, lung adenocarcinoma; SHMT2, serine hydroxy methyltransferase 2.

Abbreviation: LN, lymph node.

^∗^ *p* < 0.05.

**Table 2:** Univariate analysis for the overall survival of LUAD patients.

Variable	Cases (*n* = 113)	Overall survival		*p* value
		Mean ± SD (months)	5 year (%)	
Sex				0.121
Female	37	66.8 ± 3.8	78.9	
Male	76	61.3 ± 3.2	64.3	
Age (years)				0.024^∗^
≤ 60 years	49	71.6 ± 3.5	75.9	
> 60 years	64	58.3 ± 2.9	64.6	
Smoker				0.481
No	50	66.6 ± 3.6	77.0	
Yes	63	61.6 ± 3.1	64.3	
Differentiation				0.447
Well	39	60.9 ± 4.2	67.6	
Moderate	47	68.4 ± 3.5	79.3	
Poor	27	58.1 ± 4.8	53.1	
Tumor size				0.193
≤ 3.0 cm	72	67.4 ± 3.2	74.6	
> 3.0 cm	41	59.3 ± 3.5	60.2	
LN metastasis				< 0.001^∗^
Negative	64	72.7 ± 3.1	81.4	
Positive	49	53.4 ± 3.1	54.8	
TNM stage				0.003^∗^
I-II	53	70.3 ± 2.7	83.6	
III-IV	60	58.3 ± 3.4	55.9	
SHMT2 expression				0.006^∗^
Low	57	68.9 ± 2.7	80.0	
High	56	57.9 ± 3.6	59.3	

*Note:* LUAD, lung adenocarcinoma; SHMT2, serine hydroxy methyltransferase 2.

Abbreviations: LN, lymph node; SD, standard deviation.

^∗^ *p* < 0.05.

**Table 3:** Multivariate analysis for the overall survival of LUAD patients.

Variable	Hazard ratio	95% CI	*p* value
Age (vs. ≤ 60 years)	2.424	1.192–4.929	0.014^∗^
TNM stage (vs. I-II)	2.760	1.233–6.178	0.014^∗^
SHMT2 expression (vs. low)	2.120	1.027–4.376	0.042^∗^

*Note:* LUAD, lung adenocarcinoma; SHMT2, serine hydroxy methyltransferase 2.

^∗^ *p* < 0.05.

## Data Availability

The data that support the findings of this study are available from the corresponding author upon reasonable request.
